# Iron Regulatory Mechanism IRE/IRP-like in Two Protozoa of Importance to Human Health, *Entamoeba histolytica* and *Giardia duodenalis*

**DOI:** 10.3390/pathogens15010057

**Published:** 2026-01-07

**Authors:** Jesús Gabriel León-Beltrán, Sarita Montaño, Rossana Arroyo, Daniela Estrada-Ramírez, Nidia León-Sicairos, Adrián Canizalez-Román, María Angélica Sánchez-González, José Antonio Garzón-Tiznado, Claudia León-Sicairos

**Affiliations:** 1 Programa Regional del Noroeste para el Posgrado en Biotecnología de la Facultad de Ciencias Químico-Biológicas, Universidad Autónoma de Sinaloa, Av. de las Américas y Josefa Ortíz (Cd. Universitaria), Culiacán 80030, Mexicommontano@uas.edu.mx (S.M.); daniela.estram@gmail.com (D.E.-R.); nidialeon@uas.edu.mx (N.L.-S.); angelicasanchez.fcqb@uas.edu.mx (M.A.S.-G.); garzon24@uas.edu.mx (J.A.G.-T.); 2 Departamento de Infectómica y Patogénesis Molecular, Centro de Investigación y de Estudios Avanzados del Instituto Politécnico Nacional (Cinvestav), Av. IPN No. 2508, Colonia San Pedro Zacatenco, Mexico City 07360, Mexico; rarroyo@cinvestav.mx

**Keywords:** *Entamoeba histolytica*, *Giardia duodenalis*, iron, regulation, IREs

## Abstract

Protozoa use iron to grow, feed, and cause harm through elaborate mechanisms to obtain it from the host. In addition, expression of virulence genes is affected by iron. In *Entamoeba histolytica,* the parasite that causes amoebic dysentery and complications in human organs, our group have previously reported the presence of an IRE/IRP-like (Iron Responsive Element/Iron Regulatory Protein) mechanism. *Giardia duodenalis* is another parasite of medical interest that causes giardiasis, including nutrient malabsorption syndrome and dysbiosis, among other complications, such as anemia in children with giardiasis. Moreover, expression of many putative giardial virulence factors by free-iron levels has been reported. Recently, we have reported stem-loop structures in some mRNAs coding virulence proteins from both parasites. However, much remains to be studied about the role of iron in pathogenesis. In this review, we summarize several aspects of gene expression regulation by iron in these protozoa as well as an iron regulatory mechanism in *E. histolytica* and discuss the possibility of an iron regulatory IRE/IRP-like mechanism in *G. duodenalis.*

## 1. Introduction

Protozoan parasites are responsible for human mortality and morbidity, causing a significant health and economic burden worldwide. Some of them have complex life cycles in multiple hosts and must initiate complex developmental programs in response to environmental cues including stress and transition to different hosts or their defenses. Many protozoan parasites use antigenic variation to elude the host immune response. Two protozoa responsible for intestinal diseases with great impact on public health worldwide are *Entamoeba histolytica* and *Giardia duodenalis*, causative agents of amoebiasis and giardiasis, respectively. Both parasitoses have great epidemiological and clinical importance due to their high morbidity and pathogenicity [1,2]. Amoebiasis is the second leading cause of death from parasitic disease worldwide. It is a major public health problem due to poor sanitation that contaminates drinking water and food with feces [3,4]. *E. histolytica* causes up to 100,000 deaths worldwide each year. Human amoebiasis is mainly characterized by local dysentery (bloody intestinal diarrhea) and in extreme cases invasive extraintestinal disease, including peritonitis and liver, pulmonary, and brain abscesses [5,6]. Giardiasis is caused by the flagellate protozoan parasite *G. duodenalis*, the most common protozoal infection in humans. In fact, Giardia has eight assemblies ranging from A to H; A and B infect humans, while C to H assemblies can infect domestic animals such as dogs and cats [7]. *G. duodenalis* is an important cause of waterborne and foodborne diarrhea, day-care center outbreaks, and traveler’s diarrhea. The disease is included in the World Health Organization (WHO) neglected diseases initiative owing to its burden and association with poverty [8]. This parasite is responsible for 280 million symptomatic infections per year. The outcome of giardiasis can vary: the infection may remain asymptomatic or lead to severe and sometimes persistent symptoms such as irritable bowel syndrome and dysbiosis [7,8,9,10,11,12].

Iron is an essential element for all living organisms and functions as a cofactor in many biochemical activities, including DNA synthesis, oxygen transport, and cellular respiration. Iron deficiency can cause cellular death, whereas iron excess is potentially toxic causing oxidative stress [13]. Therefore, the iron concentration in the cell must be carefully regulated. The IRE/IRP system (Iron Responsive Element/Iron Regulatory Protein) is an iron regulatory mechanism at the posttranscriptional level that has been widely studied in higher eukaryotes. It specializes in maintaining iron homeostasis in cells for optimal functioning. In recent years, this study has been extended to protozoan parasites [14,15,16] that use iron-regulated virulence genes to obtain iron from the host to feed, survive, and cause damage. Iron is an important factor for *E. histolytica* growth, adherence, and cytotoxicity, and it modulates its gene expression [17,18]. Iron is also an essential component of *E. histolytica* enzymes [19], such as iron superoxide dismutase (FeSOD) and hemoglobinases [4,20,21]. Our research group has reported IRE-like structures in the untranslated-regions (UTRs) from mRNA coding virulence factors. These mRNA stem-loop structures specifically bind to human IRP and amoebic cytoplasmic proteins in low iron conditions, suggesting the presence of an IRE/IRP-like regulatory system in *E. histolytica* [4,16]. Recently, in *G. duodenalis* an iron effect in the expression of many putative giardial virulence factors in RNAseq experiments has been reported [22], as well as the presence of stem-loop structures in some of the mRNA encoding virulence factors [23]. In other protozoans such as *Trichomonas vaginalis,* the causal agent of trichomonasis, the most common sexually transmitted infection, the presence of IRE-like structures in some iron differentially regulated mRNAs [14,15,24], as well as atypical RNA-binding proteins under iron-restricted conditions that bind to these structures have been reported [25]. Despite having found stem-loop structures in these three parasites with similar motifs, an IRP/aconitase homolog sequence has not been found in their genomes, suggesting the presence of multifunctional proteins with RNA-binding functions, such as Tvactinin-3 and TvHSP70 in *T. vaginalis* [14,15,24,25]. Thus, much remains to be understood about the iron regulatory mechanism in these parasites. In this review, we highlight the importance of iron gene regulation and discuss the presence of a putative iron regulatory mechanism mainly in *E. histolytica* and *G. duodenalis*. In addition, *in silico* analysis related to possible RNA–protein interactions in Giardia is presented and discussed. It is the protozoan with the fewest studies on this subject, in spite of the fact that in this parasite the growth and expression of several virulence genes are also affected by iron.

## 2. Mechanisms of Pathogenicity and Virulence Factors in *E. histolytica*

For *E. histolytica* to cause disease, multiple mechanisms are needed. Trophozoites adhere to host mucous and epithelial cells and secrete multiple cysteine proteases (CPs), which degrade mucin and extracellular matrix, and they also kill host cells through a contact-dependent process. This parasite also invades and phagocytizes red blood cells and nucleated host cells [26]. The principal mediator of adherence is the amebic *Gal/GalNAc* lectin which participates in adherence and cytotoxicity of trophozoites to mammalian cells, erythrophagocytosis, and liver abscess formation in hamsters [27,28,29,30].

Other molecules involved in the adhesion process of *E. histolytica* have been reported, such as cysteine and serine proteases and the EhCPADH complex [31,32], lectin 220, a protein enriched with lysine and glutamic acid (KERP1), Pyruvate: ferredoxin oxidoreductase (PFO), and a rhomboid protein (EhROM) [5,33].

*E. histolytica* excretory-secretory products (ESPs) may play critical roles during invasion. The non-pathogenic Rahman strain showed reduced secretion of Enolase 1, alcohol dehydrogenase, transketolase, malic enzyme, phosphoglucose mutase, superoxide dismutase (SOD), and PFO. Some of these proteins control carbohydrate metabolism but may have other moonlighting effects [34,35].

Most of the antioxidant activity of *E. histolytica* against reactive oxygen species (ROS) generated by neutrophils and macrophages from the host is dependent on two proteins, a peroxiredoxin and the iron-dependent superoxide dismutase (FeSOD) [36]. Overexpression of peroxiredoxin in the avirulent Rahman strain restores its resistance to oxidative stress and its ability to cause colitis [37]. High-oxygen-exposed *E. histolytica* lysates showed up-regulation on mRNAs of the thiol-dependent peroxidase (Eh29), FeSOD, and heat shock protein 70 (HSP70) [38].

CPs of *E. histolytica* in addition to participating in adherence to enterocytes are important for combating host defenses and favor the infection process. The expression of six CPs *(EhCP1–EhCP6*) under iron-restricted conditions was increased [17,18]. The most highly expressed CPs are *EhCP1*, *EhCP2,* and *EhCP5*, which are responsible for ~90% of enzymatic activity [4,17,18].

Most virulent *E. histolytica* trophozoites can invade and produce liver abscesses. These trophozoites show up-regulation of genes encoding heat shock proteins, which are molecular chaperones that allow cells to survive during stress by maintaining proper protein folding [39]. When invasive disease occurs by an *E. histolytica* infection, there is a potent cytotoxic activity. The parasite kills and ingests host cells in a contact-dependent manner using Gal/GalNAc lectins. Tissue damage is also augmented by parasite proteases and inflammatory host immune reactions [34,40]. *E. histolytica* secretes macrophage migration inhibitory factor (EhMIF) that promotes mucosal inflammation, resulting in an increased production of matrix metalloproteinases that break down extracellular matrix in the gut to promote cell migration. These changes allow immune cells infiltration, generating ROS to kill the parasite. Free radicals are responsible for collateral tissue damage while *E. histolytica* may be able to evade the immune response [41]. After degradation of epithelial cells, the parasite navigates through extracellular matrix for dissemination to the extra-intestinal sites. Moreover, *E. histolytica* requires glycosidases and proteases for the basement membrane disintegration and entry into the circulation [40].

## 3. Mechanisms of Pathogenicity and Virulence Factors in *G. duodenalis*

*Giardia* is a non-invasive parasite which infects the small intestine and colonizes the lumen and epithelial surface. Trophozoites attach to epithelial cells and induce shortening of microvilli. The parasite targets specific signaling networks that activate apoptosis, leading to the loss of intercellular junctions, barrier function, and cytoskeletal rearrangement, which contribute to diarrhea [42]. Some virulence factors have been identified in the parasite, such as the adhesive disk and the four flagella [43,44]. The giardial ventral disk mediates reversible parasite attachment to the host intestinal microvilli. This attachment allows *Giardia* to resist peristalsis and remain in the gut [44,45,46]. The disk is primarily composed of ankyrins and novel hypothetical proteins that have no homology to proteins outside of *Giardia* species. Early biochemical studies reported the presence of proteins termed “giardins” [46,47]. There are different giardin classes of proteins (alpha-, beta-, gamma-, and delta-giardins) [48]. Recombinant alpha-1 giardin binds thin sections of human small intestine to the apical surface of epithelial cells but also other cellular structures rich in glycosaminoglycans. It has been suggested that this protein may play a role in early host–parasite interactions [48].

Virulence factors are necessary in giardiasis pathogenesis. The contact between *G. duodenalis* and the small intestine epithelium might induce the secretion of some of these factors, such as CPs, variant surface-proteins (VSPs), ESPs, tenascins, and high cysteine membrane proteins (HCMPs). Attached parasites can counteract host defenses by up-regulating their protein ubiquitination and antioxidant responses [22,49].

Although some molecules implicated in the adhesion mechanism remain incompletely understood, some attachment models have been established, such as ligand-specific, ligand-independent, and suction-mediated mechanisms [50]. However, none of them could completely explain the attachment of *Giardia.* Studies indicate that host–parasite interactions are stimulatory, inducing expression of parasite factors, which have limited or no expression in axenic culture alone. Parasites exposed to host soluble factors triggered up-regulation of membrane and secreted proteins, including cathepsin-B precursor, cystatin, tenascins, and VSPs, promoting a motile population, potentially to continue its migration through the gut to a less hostile environment. Trophozoites attached to host cells respond by up-regulating intracellular pathways involved in disappearance of ROS, anticipating host defense response [51].

Parasites produce ESPs that include secreted proteins, released surface proteins, and extracellular vesicles (EVs). These ESPs affect gene expression, signaling, metabolism, secretion, and immune responses of intestinal epithelial cells (IECs) and parasite attachment induce stronger and more complex responses [52]. It has been reported that *Giardia* EVs have relevant virulence factors such as arginine metabolizing enzymes, cathepsin-B, and VSPs, as well as giardins, katanin, and ankyrin repeat proteins, which have prominent adhesion roles [53]. VSPs can undergo antigenic variation, their size ranging from 60 to 180 kDa. The *vsp* gene repertoire has been estimated at 270 to 300, about 4% of the genome. These proteins appear to play a role in immune evasion and protect the trophozoites from intestinal proteases. The mechanism for switching the expression of one *vsp* gene to another remains to be determined. Although, there is evidence supporting two mechanisms: lysine acetylation, which limits expression to even one allele of four and an RNAi system that limits expression at a posttranscriptional level [54,55].

*G. duodenalis* lacks some of the conventional mechanisms of the oxidative stress management system, including SOD, catalase, peroxidase, and glutathione cycling, which are present in most eukaryotes [56]. However, it possesses alternative antioxidants, such as NADH oxidase and a flavoprotein, which can detoxify oxygen to form water; SOR (superoxide reductase) that converts superoxide to hydrogen peroxide; some peroxiredoxins with the ability to detoxify hydrogen peroxide to form oxygen and water; and low molecular weight thiols. It has also been reported that on albendazole-resistant *Giardia,* NADH oxidase, and peroxiredoxin are overexpressed. Moreover, the role of pyruvate against oxidative stress in *G. duodenalis*, exerting antioxidant activity, was reported in some studies [57,58,59].

There are still questions around *G. duodenalis* adhesion and the molecules that participate in this mechanism. Our research group identified some possible ortholog adhesin proteins using the tool BLAST in *Giardia*DB website; protein sequences for adhesin from *E. histolytica* and *T. vaginalis* were used to perform the BLAST analysis with the most recent giardial genome sequence reported [60]. Among the most important sequences obtained from *Giardia*DB database were two pyruvate, flavodoxin oxidoreductase (GL50803_0017063, GL50803_00114609) that share homology with PFO from both *E. histolytica* and *T. vaginalis*, a malate dehydrogenase (GL50803_0014285) sharing homology with *T. vaginalis* AP65 (adhesin protein 65), and some cathepsins B and cathepsins L that share homology with *E. histolytica* cysteine proteases (*EhCP1*, *EhCP2,* and *EhCP5*). Along with BLAST from *Giardia*DB, an analysis with ClustalW was performed to obtain the similarity between the sequences from *Giardia* genome and the other parasites (Table 1).

Another of the main mechanisms by which *G. duodenalis* can invade and produce illness is the releasing of substances with cytotoxic effects against the host, promoting cytoskeleton and cell membrane degradation, metabolic functions and compound synthesis interruption, and cell division damage. These kinds of substances are regulated by iron in *T. vaginalis* [14,61,62,63,64] and *E. histolytica* [65,66,67,68]. Thus, we took several of these sequences from *T. vaginalis* and *E. histolytica* as probes to search for possible orthologs in the Giardia genome. When TvCP4, TvCP12, TvCP30, TvCP39, and TvCP65 sequences were used as probes in BLASTp, several possible orthologs of cytotoxicity factors in *G. duodenalis* proteome were found, among which cathepsin precursors and other cysteine proteases such as cathepsin B and cathepsin L proteins with 18–22% identity were found (Table 2). It is of particular interest to the cathepsin B precursor (GL50803_14019), also known as *giardipain-1*. It is the most expressed cysteine protease in this parasite, which is able to degrade cell–cell junctional components and induce apoptotic damage in epithelial cells [49,69]. As for *E. histolytica* EhCP1, EhCP2, and EhCP5 which were used as probes [4], they also showed homology with *G. duodenalis*’s cathepsins B and L with 18 to 22% identity (Table 2). Remarkably there are some repetitions in the homolog proteins obtained, such as cathepsin B: GL50803_0014019 giardipain-1 [49] and some other cathepsins such as GL50803_0016160. GL50803_0014983, GL50803_0010217, GL50803_0016380, and GL50803_0016468 share homology with multiple CPs from other protozoans, which may suggest that they are virulence factors relevant in *G. duodenalis*.

## 4. Iron Regulation in Protozoa

Iron is an essential metal ion for all organisms. It is a known regulator of virulence genes in many bacteria and pathogenic protozoa. Iron is an essential constituent of many proteins in pathogens, such as metabolic, antioxidant enzymes, and virulence factors [70,71]. Tight regulation of iron in mammalian hosts presents an obstacle to invading pathogens. Therefore, protists have evolved efficient mechanisms to exploit host iron sources. Thus, this host–pathogen competition for iron is a deciding factor in the success of an infection.

### 4.1. E. histolytica

Iron is a necessity for *E. histolytica* trophozoites; the parasite uses diverse human proteins, such as hemoglobin, holo-lactoferrin, holo-transferrin, and ferritin as sources of iron. It is an important factor for parasite growth, adherence, and cytotoxicity. It also modulates gene expression [4,16,72]. Some of these genes are *EhCP1–EhCP6*, hemoglobin-binding proteins (*Ehhmbp45* and *Ehhmbp26*), *FeSOD*, *actin*, and *Acyl-CoA synthetase* [4]. Iron concentration also influences cytoadherence and cytotoxicity [17]. In these studies, parasites were cultured in medium prepared without ammonium ferric citrate, treated with 0.5 g per 100 mL Chelex-100 which was removed by filtration, to obtain iron-restricted conditions (6.5 µM). For the iron-rich medium, the BI-S-33 medium was prepared to a final concentration of 250 µM ammonium ferric citrate [4,16,17,72]. There is reported data suggesting the presence of a posttranscriptional iron regulatory IRE/IRP-like mechanism in *E. histolytica*, supported by specific RNA–protein interactions in RNA electrophoretic mobility shift assays [4,16]. Some key iron-dependent enzymes in amoebas include acetaldehyde/alcohol dehydrogenase-2, ferredoxin, diaphorase, and SOD. Fe-S proteins are also important. They are necessary in energy metabolism and electron transfer in the parasite. *E. histolytica* can cleave ferritin into several fragments. Three neutral cysteine proteinases (100, 75, and 50 kDa) [20] were observed to degrade ferritin in culture extracts. In addition, amoebae quickly internalized ferritin via clathrin-coated vesicles. *E. histolytica* trophozoites have the capacity to phagocytose and lyse red blood cells [73] with the purpose of accessing the hemoglobin. Once this protein is available to amoebae, it is cleaved by hemoglobinases, releasing the haem group and amino acids, which are used by the parasite as iron and energy sources for growth. In *E. histolytica in vitro* cultures have been reported that Lf protein can be either amoebicidal or used as an iron source for growth, depending on the Lf saturation [20]. It has been suggested that *E. histolytica* uses five pathways to access the host iron: hemophore-like proteins that bind free heme; several transporter families up-regulated in iron-limited conditions; internalizing hololactoferrin, holotransferrin, and ferritin via receptor recognition pathway; and performing phagocytosis of whole erythrocytes and using both ferric and ferrous iron [71].

### 4.2. G. duodenalis

There is almost no information regarding iron’s direct relationship with *G. duodenalis*. Recently, a few reports about some genes that are differentially regulated by iron concentration *in vitro* and stem-loop structures in some mRNA of virulence proteins have been reported [22,23]. Peirasmaki et al. cultured the Giardia trophozoites in a TYDK medium without ferric ammonium citrate and 50 µM of 2-2′ bipyridyl to obtain iron-restricted parasites [22], while our group cultured the trophozoites in a TYI-S-33 medium without ferric ammonium citrate and the Chelex-100 resin (to obtain a 7.7 µM final concentration of iron) as has been reported [16,23]. In addition, based on clinical cases, it appears that infections by the parasite turn out to be related to anemia or iron deficiency, resulting in an improvement of these symptoms when the infection is treated with metronidazole [11,74,75,76]. Thus, a clear relationship with *G. duodenalis* and iron exists but is not fully understood.

*Giardia* trophozoites can damage the IECs leading to the malabsorption of water, electrolytes, glucose, and maldigestion due to the loss of digestive enzymes [77,78]. Studies related to trophozoite attachment causes damage to the intestinal epithelial cells [79] and release cysteine proteases, tenancins, metabolic enzymes, HCMPs, and VSPs [10,51,52,80,81,82]. In addition, these studies showed that HCMPs are localized in the trophozoite plasma membrane and are regulated by histone acetylation and levels of free iron in the culture medium [22,82]. The transport of iron within cells of parasitic protists is poorly understood. In *G. duodenalis* there is a lack of information [77]. Recently, our research group performed a BLASTp analysis to find potential proteins homologs in *G. duodenalis* genome using *T. vaginalis* ZIP proteins as probes, which have been suggested to participate in iron and zinc uptake [83]. In addition, a protein from *E. histolytica* Rab7A was used as a probe, due to its role in endocytosis from early phase holo-transferrin [84]. TvZIP2 and TvZIP4 proteins shared around 16 and 17% identity with GL50803_006664 zinc transporter protein (Table S1 on Supplementary Material File S1). On the other hand, *E. histolytica* Rab7A protein matched with Rab2a (GL50803_005567), Rab2b (GL50803_0016636), and Rab1a (GL50803_009558) proteins from *G. duodenalis* with 26–27% identity (Table S1 on Supplementary Material File S1). These alignments may suggest that these proteins may be involved within an iron uptake mechanism in *G. duodenalis*, similar to that one present in *T. vaginalis* and *E. histolytica*; however, further investigation is needed to support this hypothesis.

### 4.3. Other Protozoa

*Trichomonas vaginalis*, the protozoan responsible for the sexually transmitted infection trichomoniasis, has high iron requirements. Iron is essential for its metabolism, division and survival [85]. Under iron deficiency *T. vaginalis* exhibits slow-growing or arrested-growth phenotype since the parasite utilizes iron-dependent metabolic systems to generate energy. The hydrogenosomal energy metabolism of *T. vaginalis* is upregulated by iron; however, the regulation of glycolysis associated with iron availability remains unclear [86,87].

*T. vaginalis* utilizes multiple sources of iron: lactoferrin, heme, and hemoglobin. The parasite has multiple iron uptake systems. It has a 136 kDa receptor for binding the host holo-lactoferrin; other receptors bind hemoglobin, heme, and cytochrome C, using AP65 and AP51 adhesins as heme- and hemoglobin-binding proteins [88].

Iron modulates the expression of several virulence factors in *T. vaginalis*, including adhesins and CPs [24]. Iron also differentially modulates some properties in *T. vaginalis* such as hemolysis, induction of apoptosis in the host cells, immune evasion, cytotoxicity, and cytoadherence [Adhesin protein 65 (*ap65*) iron inducible transcription] just by repression or induction of the cysteine proteinases expression [64,88]. The *T. vaginalis ap65* gene is regulated at the transcriptional level by an iron responsive promoter that includes an iron-responsive DNA element overlapping with 3′-MYB-like protein-binding sequence [89]. It has been reported that iron depletion in *T. vaginalis* reduces the protein synthesis and cell density, although it increases the expression of lactoferrin-binding receptor [90]. This parasite resides in an environment with fluctuating iron availability. Thus, mechanisms to respond to iron limitations are important for its adaptation and survival.

*Plasmodium falciparum,* the causal agent of malaria, is an obligate intracellular parasite that relies on bioavailable iron to meet its nutrient requirements [91]. *P. falciparum* has an intra-erythrocytic proliferation and uses iron for pyrimidine and heme synthesis; the parasite also needs to balance its need for iron and the cytotoxicity of the metal [92]. Interestingly, *P. falciparum* metabolizes hemoglobin to acquire amino acids, but it does not utilize that iron. Instead, it uses the cytoplasmic iron pool of red blood cells, iron that is not incorporated into hemoglobin or stored as ferritin [93]. This adds complexity to the relationship between the parasite and iron and may explain the existence of the *P. falciparum* IRP reported by Loyevsky et al., 2001 [94]. There is still a lot of unknown data about this parasite’s iron biology. To date, the main studies related to iron’s effect on virulence have been focused mainly on these protozoans.

## 5. The IRE/IRP-like Regulatory System in Protozoan

Iron is essential for all living organisms. Because both the deficiency and the iron overload are harmful, iron absorption, concentration, and redox status should be carefully regulated [95,96,97,98]. The post-transcriptional regulation by the IRE/IRP system is the most studied iron regulatory mechanism in mammalian cells. The IRE/IRP system involves IREs (Iron Responsive Elements) and IRPs (Iron Regulatory Proteins), where under low iron conditions IRPs bind IRE structures, which are stem-loop secondary RNA structures present in the 5′ or 3′ UTR of iron-regulated mRNA. Under low-iron conditions, IRPs modulate the expression of proteins involved in iron metabolism by binding to conserved IREs. The regulatory outcome depends on the position and context of the IRE in the mRNA: an IRP bound to a 5′-UTR IRE represses translation, whereas an IRP bound to a 3′-UTR IRE can indirectly activate translation via the suppression of mRNA degradation. At high iron concentrations, IRP1 acts as a cytosolic aconitase enzyme, whereas IRP2 is degraded. The dual roles of IRP1 link gene regulation in iron homeostasis to the sensing of intracellular iron levels and oxidative stress [99]. The coordinated regulation of the IRE/IRP network enables cells to respond to multiple signals of iron availability and demand in a balanced manner [100,101,102,103].

IREs contain two regions necessary for IRP binding: the consensus CAGUGN loop sequence is most frequently found, and UAGUAN is less common [99]. The contiguous stem sequence, containing either a conserved C nucleotide five bases upstream of the CAGUGN sequence, creates a bulge in the hairpin, or a UGC/C loop-bulge [104]. Numerous studies have reported RNAs with non-canonical loop sequences that still interact with IRPs, even though many of those sequences are mutants of the canonical IREs [105] (Table 3).

This system was first described in humans [95,96,97,98] on mRNAs that code for proteins involved in iron homeostasis. Interestingly, some IRE-like structures were described in the parasite *T. vaginalis* that were recognized by human IRPs and atypical RNA-binding trichomonad proteins [14,15,24,25]. Moreover, some IRE-like structures were also described *in silico* for *E. histolytica* by our research group [16] (Table 3). In addition, certain *E. histolytica* mRNAs contained IRE-like structures in both the 5′- and 3′-UTR. However, dG (Gibbs free energy or free enthalpy, where, in this case a reduction in dG (negative dG) is a necessary condition for spontaneous structure formation) could help predict which structure is more stable, as well as the up- or down-regulation by iron.

*P. falciparum* possesses an IRP-like protein that binds consensus IRE sequence and a putative plasmodial IRE; the binding is iron-regulated, and the IRP-like protein has aconitase activity. This regulation is very similar to IRE/IRP system of higher eukaryotic cells [94]. However, there are no more recent studies related to other IRE-like in this protozoon.

Discussion around IRE-like structures not having consensus motifs in the loop as canonical IREs; for example, Senoura et al. 2020 reported that rice aconitase OsACO1 protein potentially has RNA-binding activity with plant ACO-interacting RNA element (PAIR), similar to mammalian IRE, raising the possibility that OsACO1 senses iron status as IRP1 [106]. These PAIR structures conserve a GGUGG motif within the loop, which is also different from consensus motifs reported in IREs. The PAIR-OsACO1 interaction mirrors an IRE-IRP system. Although the RNA structures do not share the consensus motifs, both have proven to be functional [106,107]. This may also be true in other organisms with IRE-like structures.

Furthermore, the presence of some residues was reported for canonical [108] and protozoan IRE-like structures [15] by the Zuker mfold program [109], while iron modulation was reported for some mRNAs. Moreover, the stem-loop structure analysis showed the presence of atypical IREs. Remarkably, the GUU/UUG protozoan-specific motif and a new motif AUU/AUUU were observed in almost all mRNAs analyzed. This new motif could be an amoebic-specific motif. Likewise, all the analyzed structures showed the presence of several motifs. Surprisingly, these sequences were also found in the stem structure, suggesting a possible interaction between an amoebic IRP-like protein and this stem sequence [16].

**Figure 1:**
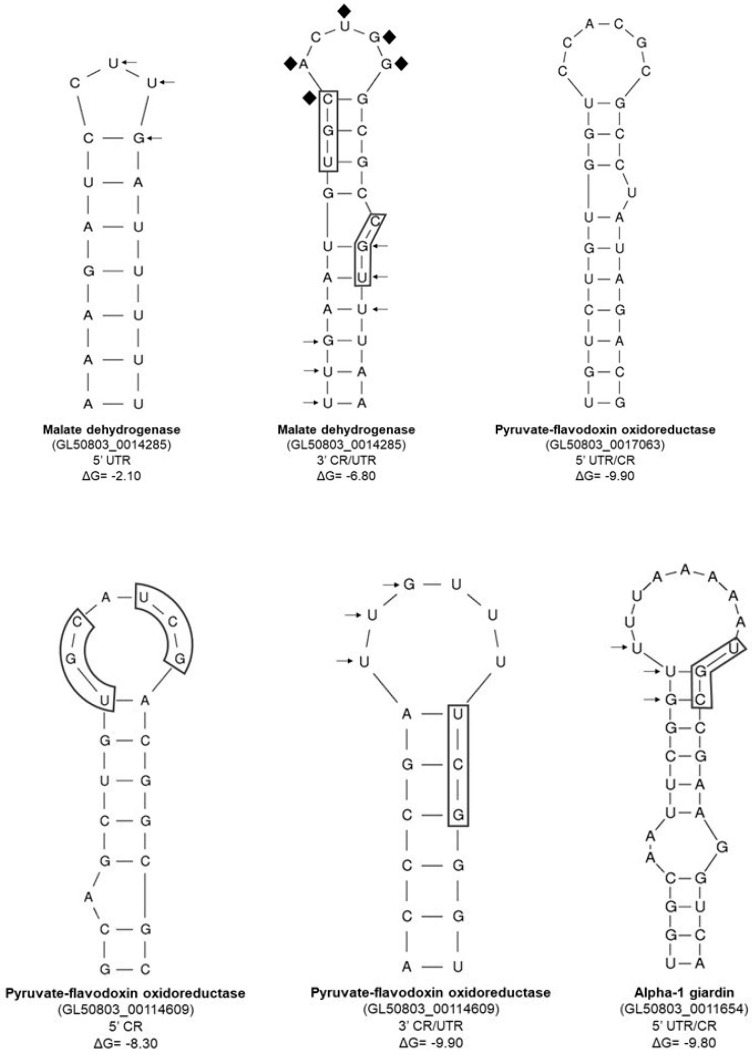
*G. duodenalis* IRE-like structures on mRNAs of adhesin homologs. The arrows indicate the GUU/UUG-protozoa-specific motif [15]. Boxes indicate the CGU motif. Diamonds indicates the sequence similar to the canonical CAGUGN of higher eukaryotes [108]. CR, coding region. dG, Gibbs free energy or free enthalpy; in this case, a reduction in dG (negative dG) is a necessary condition for spontaneous structure formation.

Given the evolutive closeness that *G. duodenalis* holds with amoebal and trichomonal parasites, our research group performed an *in silico* analysis using the Zuker mfold Software [109] in order to search for possible IRE-like structures in the mRNAs of the putative giardial adhesin orthologs obtained by the BLAST analysis and alpha-1 giardin which may have an important role in adhesive mechanisms [110] (Figure 1). The theoretical and *in silico* analysis were carried out as has been described previously [16], based on regulatory elements reported [111,112,113]. Some of the IRE-like structures found in these genes are within the coding region of the mRNAs, as has been reported in *T. vaginalis* structures [14], possible due to the short UTR regions of *G. duodenalis* genome. The IRE-like structures found in our study may be considered non-canonical because the consensus loop sequence of human IRE structures (CAGUGN) was not found. Nevertheless, these hairpins have some specific motifs reported in parasites (GUU/UUG and AUU/UUA) [15,16]. In addition, some of these structures are also predicted by SIREs web server, despite the SIREs web server being based on canonical IREs (human ferritin-IRE) [108].

**Figure 2:**
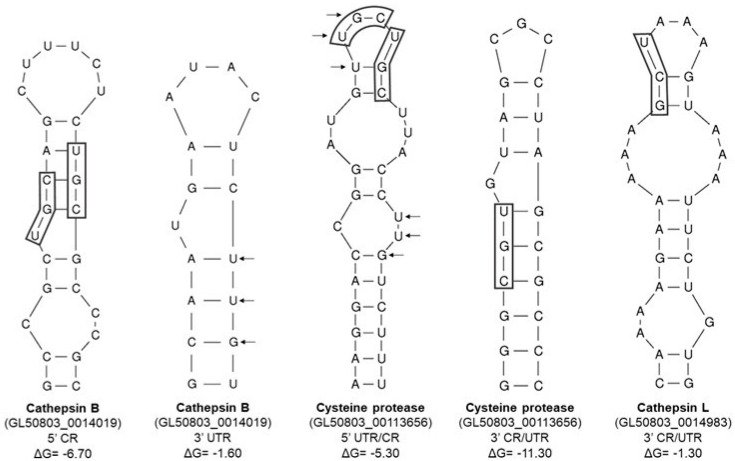
Prediction of stem-loop structures of mRNAs coding for possible orthologs from CP cytotoxicity factors of *G. duodenalis*. The arrows indicate the GUU/UUG-protozoa-specific motif [15]. Boxes indicate the CGU motif [104]. CR, coding region. dG, Gibbs free energy or free enthalpy; in this case, a reduction in dG (negative dG) is a necessary condition for spontaneous structure formation.

Moreover, these possible orthologs from cytotoxicity factors (CPs) mentioned before (Table 2) were also analyzed by the Zuker mfold Software [109] and several stem-loop structures were found in the UTRs as well as coding regions (Figure 2). Although, these were not identical to the typical IREs found in higher eukaryotic organisms [108]. The GUU/UUG-protozoa-specific motif was identified in some of these giardial stem-loop structures [15] (Figure 2). It is important to mention that Piccinelli and Samuelsson, 2007 [114], reported a bioinformatic analysis of mRNA sequences for more than 100 novel sequences for ferritin, mitochondrial aconitase, transferrin receptor, ferroportin, and DMT1 from different species [114]. These sequences formed IRE-type structures with the CAGUGN sequence in the loop and, in some IREs, a bulge with the UGC/C nucleotides in the stem with an unpaired cytosine is present. The IRE-like structures from *G. duodenalis* bioinformatic analysis in this study, despite lacking the canonical CAGUGN sequences in the loop, in some structures, the UGC/C motif was observed (Figure 1 and Figure 2).

To validate these bioinformatic results, interactions between these RNA hairpins and cytoplasmatic proteins from trophozoites grown under different iron conditions are being performed in our lab. Recently, bioinformatic analyses were carried out within the *G. duodenalis* genome and several IRE-like structures were found in the UTRs from different virulence factors that are modulated by iron, suggesting the presence of an iron regulatory mechanism similar to the IRE/IRP system of higher eukaryotes [23]. Thus, we hypothesized that a similar mechanism to the canonical IRE/IRP system could also exist in *G. duodenalis*.

**Table 3:** IRE-IRP comparative data between mammalians and protozoans.

**IREs**	**Canonical IREs**	**Non-Canonical IREs**
	*Homo sapiens Ferritin* IRE: UUCCUGCUUCAACAGUGCUUGGACGGAA [114]	*P. falciparum* IRE-1: AACUUAUAAAGUUAUAUAAUU [115]
	*H. sapiens Tfr1* IRE: UAUUUAUCAGUGAGCAGUGCCUCACUAUAAAUG [98]	*P. falciparum* IRE-2: UUCUUGUUAAGUUGAACAAAA [115]
	*H. sapiens Ferroportin* IRE: AACUUCAGCUACAGUGUUAGCUAAGUU [98]	*T. vaginalis cp4*: UCGUUCAGGCACAUGAGCAGA [15]
	*H. sapiens* DMT1 IRE: GCCAUCAGAGCCAGUGUGUUUCUAUGGU [114]	*T. vaginalis cp12*: AACGUAUUUAAUUGAUUGCGAA [15,116]
	*H. sapiens* mACO IRE: UCAUCUUUGUCAGUGCACAAAAUGG [108]	*E. histolytica Ehhmbp26:* AAUAAAUUGAAUUAAUGUUCUCUUUAUU [16]
	*H. sapiens* eALAS IRE: GUUCGUCCUCAGUGCAGGGCAAC [108]	*G. duodenalis pfo*: ACCCGAUUGUUUUCGGGU [23]
**IRPs**	**Mammalian IRPs**	**Suggested IRP-like**
	*H. sapiens* IRP1 (Cytosolic Aconitase) 889 aa [95]	*P. falciparum*: PfIRPa 909 aa (47% homology with human IRP1) [94]
		*T. vaginalis*: TvACTN3 1129 aa (No homology with other IRPs) [25]
	*H. sapiens* IRP2 963 aa [95]	*T. vaginalis*: HSP70 659 aa (No homology with other IRPs) [116]
		*T. vaginalis*: PSP1 124 aa (No homology with other IRPs) [117]
		*G. duodenalis*: Translation Initiation Inhibitor 120aa (Shares homology with PSP1 32.5%) [This study]

Tfr1: Transferrin Receptor 1. DMT1: Divalent Metal Transporter 1. mACO: Mithocondrial Aconitase. eALAS: Erythroid Delta-Aminolevulinate Synthase. TvACTN-3: Tvactinin-3. TvHSP70: Tvheat shock protein 70. Tv-PSP1: *Trichomonas vaginalis* perchloric-acid-soluble protein. Colored letters indicate consensus motifs. CAGUG is the canonical motif for IREs, and GUU/AUU is found in non-canonical IRE-like structures in protozoans. aa: amino acid.

A lot of questions remain around the IRE/IRP system in protozoan parallel to metazoans. There are multiple reports of non-canonical IRE-like stem-loop structures within the sequence of some virulence factors of these microorganisms [4,15,16,23,116]. Some of these RNA structures interact with proteins, including human IRP and trichomonads proteins such as HSP70 and α-Actinin 3 [116]. Recently Millán-Pacheco et al., 2023 [117] reported a PSP1 protein (Tv-PSP1) in *T. vaginalis* that binds stem-loop IRE-like and ERE-like structures (eukaryotic initiation factor 5A response element stem-loop which participates in mRNA expression and stability) by RNA electrophoretic mobility shift assays (REMSA). These investigations suggest that even without canonical motifs within these RNA structures exists some type of regulation involving iron and RNA–protein interactions.

**Figure 3:**
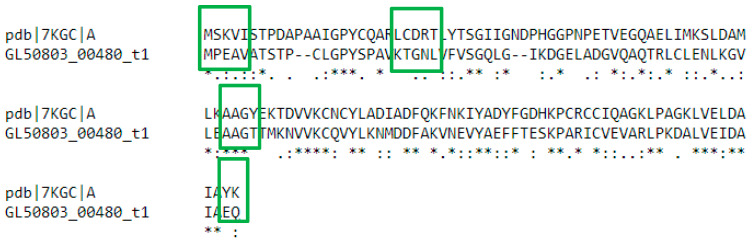
Alignment between Tv-PSP1 (PDB: 7KGC D) and translation initiation inhibitor, TII (GL50803_00480). The green squares indicate highlighted residues in RNA–protein interface of TvPSP1 and IRE-like interaction. TII (GL50803_00480) holds conserved residues in some of these regions. (*) indicate equal residues, (:) indicate highly conserved residues, (.) indicate weakly conserved residues.

We performed BLASTp analysis on the website giardiaDB to identify proteins that share homology with these IRE-like stem-loop RNA-binding proteins (PSP1, HSP70, and α-Actinin 3) in *G. duodenalis*. Our findings showed proteins with homology between Tv-HSP70 and GL50803_0088765 Cytosolic heat shock protein 70, GL50803_0017121 Bip, and GL50803_0014581 Chaperone protein DnaK HSP70 from *G. duodenalis* (Table 4). This is expected based on the nature of these proteins. Although, this does not discard the possibility of interaction between these molecules and RNA stem-loop structures. In the case of Tv-PSP1, we found 32.5% homology by ClustalW alignment with translation initiation inhibitor (TII) (GL50803_00480) from *G. duodenalis*. The alignment shows that the protein TII (GL50803_00480) has some key conserved residues that Tv-PSP1 utilizes to interact with stem-loop structures (Figure 3) [117].

**Figure 4:**
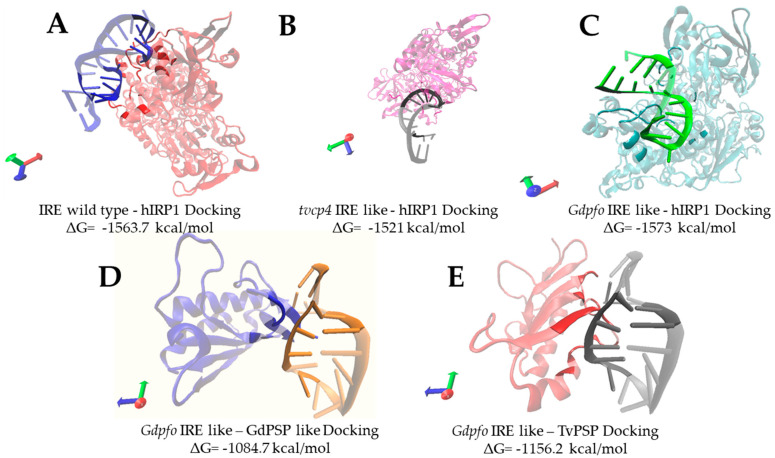
Docking models between IRE and IRE-like structures with hIRP-1 and IRP-like proteins. Highlighted regions in every model represent contact residues. (**A**). IRE Wild-type (PDB: 1NBR) is represented in blue and human hIRP1 (PDB: 2B3X) in red. (**B**). *T. vaginalis* cysteine protease 4 (*tvcp4*) IRE-like structure (simulated by simRNA2.0) represented in black and human IRP1 (PDB: 2B3X) in magenta. (**C**). *G. duodenalis* pyruvate flavodoxin oxidoreductase (*Gdpfo*) IRE-like structure (simulated by simRNA2.0 (https://genesilico.pl/SimRNAweb, accessed on 7 October 2025)) is represented in green and hIRP1 (PDB: 2B3X) in cyan. (**D**). *G. duodenalis pfo* IRE-like structure represented in gray and *G. duodenalis* PSP-like protein (GdPSP-like or translation initiation inhibitor GL50803_00480, TII) in red (modeled with I-TASSER). (**E**). *G. duodenalis pfo* IRE-like structure represented in orange and *T. vaginalis* PSP (TvPSP, PDB: 7KGC) in blue. Structures were modeled with VMD (http://www.ks.uiuc.edu/Research/vmd/, accessed on 7 October 2025) and dockings were performed using Cluspro2.0 [118,119,120,121,122,123,124,125,126,127,128,129,130].

Lastly, we have been performing several *in silico* analyses, which suggest the presence of an IRE/IRP-like system in *G. duodenalis*. Recently, based on the hypothesis that *Giardia*’s PSP-like could be a possible ortholog of *T. vaginalis* PSP [117] and bioinformatic tools to simulate RNA, we performed docking using ClusPro2.0 [118,119,120,121]. We also used PDBsum to obtain details about interactions between IRE/IRE-like structures and IRP/IRP-like. Structures were either downloaded from the Protein Data Bank (PDB) or modeled by SimRNA for RNA and I-TASSER for proteins (Figure 4) [118,119,120,121,122,123,124,125,126,127,128,129,130]. hIRP (PDB: 2B3X) and IRE wild type (PDB: 1NBR) were used as controls given that it is already experimentally reported that these molecules formed RNA–protein complexes when they interact. The *tvcp4* IRE-like structure was also used as a control, since the interaction between this IRE-like structure and hIRP was experimentally shown [14]. Here, we suggest the putative interaction between *pfo* IRE-like structure of *G. duodenalis* [23] and hIRP, TvPSP, and *G*dPSP-like by docking analysis. The docking between IRE-wild type and hIRP analysis was observed; there were eight hydrogen bonds in the stem and unpaired C typical of these structures (Figure S1 on Supplementary Material File S1, Figure 4). There were also five hydrogen bonds on the loop AGUGC (Figure 4A). This matches with the information reported by Volz in 2021 [108] that highlights the AGU region in this interaction. As to the interaction of *tvcp4* IRE-like with hIRP there were two hydrogen bonds in the stem; however, the GGCACA loop region showed most of the hydrogen bonds with 13 interactions in this region (Figure 4B and Figure S1 on Supplementary Material File S1). As for the *Giardia*’s *pfo* IRE-like structure interaction with hIRP, there were 17 hydrogen bonds around the whole structure; there were 10 hydrogen bonds in the stem and seven in the loop, and most of the nucleotides in that region participated (Figure 4C and Figure S1 on Supplementary Material File S1). The *Giardia*’s *pfo* IRE-like structure interaction with *G. duodenalis* PSP-like (TII) had most of its bonds (12 hydrogen bonds) at the stem and just one at the end of the loop (Figure 4D and Figure S1 on Supplementary Material File S1). In the *Giardia*’s *pfo* IRE-like structure’s interaction with *T. vaginalis* PSP (PDB: 7KGC), there were not as many bonds as in other dockings (eight hydrogen bonds in total, seven in the stem and one in the loop). Interestingly, some of the residues that participate in these interactions were reported by Millán-Pacheco et al., 2023 [117] as important in RNA-binding activity (Figure 4D and Figure S1 on Supplementary Material File S1).

This data suggests that control RNA–protein interactions have bonds on the loop and stem, most of them with positively charged amino acids. This behavior was also observed in the docking with *pfo* IRE-like structure and proteins tested. It is important to mention that there were some bonds at the beginning and at the end of the RNA structures (5′- and 3′-end), where *in vivo* may not be possible due to the rest of the RNA around the stem-loops. In general, the behavior observed in these dockings shows the importance of positively charged amino acids in the interactions with the RNA. Most of the bonds included this kind of residue, as expected given the negative charge of the RNA molecule. Multiple interactions were with phosphate groups of the RNA. Some neutral amino acids that form polar bonds also appear *in silico* RNA–protein interactions (Figure S1 on Supplementary Material File S1).

It is important to mention that the objective of this analysis is not to confirm or determine new IRE-like structures; there are limitations within this *in silico* analysis. Thus, further experiments such as molecular dynamics simulation and REMSA are necessary to validate these putative RNA–protein interactions predicted by docking analyses. The objective of this analysis is to suggest possible molecules that may be involved in this type of interaction. We will continue investigating the presence of the IRE/IRP-like regulation mechanism by *in vitro* studies in these protozoan parasites.

## 6. Conclusions and Perspectives

As we have discussed in this review, iron is an essential cation that can regulate some of the most important functions of protozoan, including its virulence factors. Undoubtedly, there is still work to be done to understand in a broader way the iron regulatory mechanisms employed by these protozoans.

There are more studies in *E. histolytica* than in *G. duodenalis*. Recently, studies were published corresponding to the iron effect on the expression of several proteins such as HCMPs [22] as well as *in silico* analysis showing the presence of IRE-like elements similar to those reported in *E. histolytica* in their iron-regulated mRNA [16,23]. These reports lead us to hypothesize that an iron mechanism mediated by an IRE/IRP-like regulatory system can occur in both protozoan parasites. IRE-like structures have the GUU/UUG protozoan-specific motif both in the loop and stem [15,16]. In addition, specific sequences to each protozoon have been observed [16,23]. The search for an IRP-like protein in both protozoa presents a more complex challenge, requiring advanced *in silico* and *in vitro* methodologies. This is because the proteins interacting with these secondary structures do not exhibit homology to a consensus IRP; instead, they are multifunctional proteins that also bind to RNA [24,25,116,117]. Advances in the study of an IRP in *T. vaginalis* help us predict which proteins might bind to these IRE-like proteins in *E. histolytica* and *G. duodenali*s. Currently, we are carrying out experiments to determine whether these Giardia IRE-like structures can bind IRP-like proteins, such as the recombinant human IRP and cytoplasmic proteins from Giardia trophozoites grown under different iron conditions. Elucidation of these iron regulatory mechanisms will help us to understand the biology of these parasites and, in the future, improve the diagnosis and prevention of *E. histolytica* and *G. duodenalis* parasites that continue causing major health problems worldwide. For instance, recently, it has been reported *in vivo* therapeutic efficacy of polypiridine compounds, PHN-H_2_ (with iron-binding affinity), can completely cure liver abscess following its administration to *E. histolytica*-infected hamsters [131]. Another example is the mammalian chelator protein lactoferrin with antiparasitic activity [132] and some studies with the iron-regulated ferredoxin and PFO (responsible for activation of metronidazole in hydrogenosomes) which can activate metronidazole more efficiently in iron-rich conditions [71]. In addition, it has been discussed how microbial iron uptake systems can be used for selective targets to impair iron-dependent virulence mechanisms by nutrient restriction [133]. In consequence, next-generation iron chelators, iron-regulated virulence factors inhibitors, and conventional antimicrobial agents combined exemplify new strategies that modulate iron availability or mimic iron function. A better understanding of iron–pathogen–host interactions can enable the development of next-generation antimicrobials that are both non-toxic and broadly effective. Therefore, a therapy based on the combinations of compounds is a promising alternative.

## Figures and Tables

**Table 1:** Possible orthologs from adhesion factors in *G. duodenalis* and their respective probes used for BLAST search.

Used Probe (NCBI)	Homolog Sequence from *Giardia*DB	Accession Number (*Giardia*DB)	Identity (%)
PFO *E. histolytica* (EAL51636.2)	Pyruvate-flavodoxin oxidoreductase	GL50803_0017063	38.4682
PFO *E. histolytica* (EAL51636.2)	Pyruvate-flavodoxin oxidoreductase	GL50803_00114609	26.2478
EhCP1 (Q01957.1)	Cathepsin L	GL50803_0014983	20.9524
EhCP1 (Q01957.1)	Cathepsin L	GL50803_0016380	23.1746
EhCP1 (Q01957.1)	Cathepsin B	GL50803_0014019	19
EhCP1 (Q01957.1)	Cathepsin B	GL50803_0016160	20.6081
EhCP1 (Q01957.1)	Cathepsin B	GL50803_0016779	17.4497
EhCP1 (Q01957.1)	Cathepsin B	GL50803_0016468	17.7049
EhCP2 (Q01958.1)	Cathepsin L	GL50803_0014983	22.8571
EhCP2 (Q01958.1)	Cathepsin L	GL50803_0016380	22.8571
EhCP2 (Q01958.1)	Cathepsin B	GL50803_0014019	19.6667
EhCP2 (Q01958.1)	Cathepsin B	GL50803_0016779	18.1208
EhCP2 (Q01958.1)	Cathepsin B	GL50803_0016160	18.5811
EhCP2 (Q01958.1)	Cathepsin B	GL50803_0016468	18.6885
EhCP5 (CAA62835.1)	Cathepsin L	GL50803_0014983	22.0126
EhCP5 (CAA62835.1)	Cathepsin L	GL50803_0016380	22.6415
EhCP5 (CAA62835.1)	Cathepsin B	GL50803_0014019	20.6667
EhCP5 (CAA62835.1)	Cathepsin B	GL50803_0016779	18.4564
EhCP5 (CAA62835.1)	Cathepsin B	GL50803_0016468	18.3607
EhCP5 (CAA62835.1)	Cathepsin B	GL50803_0010217	19.1419
AP65 *T. vaginalis* (AAA87406.1)	Malate dehydrogenase	GL50803_0014285	29.8025
PFO *T. vaginalis* (AAA85495.1)	Pyruvate-flavodoxin oxidoreductase	GL50803_0017063	36.3872
PFO *T. vaginalis* (AAA85495.1)	Pyruvate-flavodoxin oxidoreductase	GL50803_00114609	28.7813

**Table 2:** Possible orthologs from cytotoxicity factors (CPs) in *G. duodenalis* and their respective probes used for BLAST search.

Used Probe (NCBI)	Homolog Sequence from *Giardia*DB	Access Number (*Giardia*DB)	Identity (%)
TvCP4 (AAV98582.1)	Cathepsin B	GL50803_0014019	20.3333
TvCP4 (AAV98582.1)	Cathepsin B	GL50803_0016779	18.7919
TvCP4 (AAV98582.1)	Cathepsin B	GL50803_0016160	18.9189
TvCP4 (AAV98582.1)	Cathepsin B	GL50803_0010217	18.4818
TvCP4 (AAV98582.1)	Cathepsin B	GL50803_0016468	18.3607
TvCP4 (AAV98582.1)	Cathepsin L	GL50803_0014983	21.9672
TvCP4 (AAV98582.1)	Cathepsin L	GL50803_0016380	22.623
TvCP12 (AAS38515.1)	Cysteine protease	GL50803_00113656	20.5479
TvCP30 (CAA54437.1)	Cathepsin L	GL50803_0014983	20.8633
TvCP30 (CAA54437.1)	Cathepsin L	GL50803_0016380	18.705
TvCP39 (ABX56032.1)	Cathepsin B	GL50803_0016779	19.1275
TvCP39 (ABX56032.1)	Cathepsin B	GL50803_0016160	18.5811
TvCP65 (AAS38514.1)	Cathepsin B	GL50803_0010217	19.4175
TvCP65 (AAS38514.1)	Cathepsin B	GL50803_0015564	18.932
TvCP65 (AAS38514.1)	Cathepsin L	GL50803_0014983	22.8155
TvCP65 (AAS38514.1)	Cathepsin L	GL50803_0016380	22.8155
EhCP1 (AAA29090.1)	Cathepsin B	GL50803_0016160	20.6081
EhCP1 (AAA29090.1)	Cysteine protease	GL50803_00113656	21.9048
EhCP2 (AAA29091.1)	Cathepsin B	GL50803_0014019	19.6667
EhCP2 (AAA29091.1)	Cathepsin B	GL50803_0016468	18.6885
EhCP2 (AAA29091.1)	Cysteine protease	GL50803_00113656	21.2698
EhCP5 (CAA62835.1)	Cathepsin B	GL50803_0014019	20.6667
EhCP5 (CAA62835.1)	Cathepsin B	GL50803_0016779	18.4564
EhCP5 (CAA62835.1)	Cathepsin B	GL50803_0010217	19.1419
EhCP5 (CAA62835.1)	Cathepsin B	GL50803_0016468	18.3607
EhCP5 (CAA62835.1)	Cathepsin L	GL50803_003099	20.4403
EhCP5 (CAA62835.1)	Cathepsin L	GL50803_0014983	22.0126
EhCP5 (CAA62835.1)	Cathepsin L	GL50803_0016380	22.6415

**Table 4:** TvHSP70 and *G. duodenalis* homologous proteins.

*T. vaginalis* Protein	*G. duodenalis* Protein	Identity (%)
TvHSP70 [25]	GL50803_0088765 Cytosolic heat shock protein 70	55.0835
	GL50803_0017121 Bip	46.3746
	GL50803_0014581 Chaperone protein DnaK HSP70	30.625

The alignment was performed using ClustalW.

## Data Availability

The original contributions presented in this study are included in the article/Supplementary Material. Further inquiries can be directed to the corresponding author.

## Supplementary Materials

The following supporting information can be downloaded at: https://www.mdpi.com/article/10.3390/pathogens15010057/s1, Figure S1: interactions for each modeled RNA-Protein docking of Figure 4A–E, Table S1: Possible Iron uptake proteins homologs in *G. duodenalis*.
