# Supplementary material for: Iron Regulatory Mechanism IRE/IRP-like in Two Protozoa of Importance to Human Health, Entamoeba histolytica and Giardia duodenalis
Source: Pathogens. 2026 Jan 7;15(1):57. doi: 10.3390/pathogens15010057 (PMC12845348; doi:10.3390/pathogens15010057)
Supplement: Supplementary file 1 [file pathogens-15-00057-s001.zip › pathogens-4035236-supplementary.pdf]

pathogens

MDPI

Supplementary material of:

Review

Iron regulatory mechanism IRE/IRP-like in two protozoa of importance to human health, *Entamoeba histolytica* and *Giardia duodenalis*.

Jesús Gabriel León-Beltrán[1], Sarita Montaño[1], Rossana Arroyo[2], Daniela Estrada-Ramírez[1], Nidia León-Sicairos[1], Adrián Canizalez-Román[1], María Angélica Sánchez-González[1], José Antonio Garzón-Tiznado[1], and Claudia León-Sicairos[1*]

Supplementary Material File S1: Figure S1, interactions for each modeled RNA-Protein docking (Figure 4 A-E). This information was obtained from PDBsum analyzing each model. We represented hydrogen bonds between structures. They may be aminoacid – sugar/phosphate/N base. The RNA region is colored blue or purple to identify if the interaction occurs in the stem or loop, respectively. Aminoacids are classified by color to identify if they are positively, negatively, or neutrally charged as well as if they have an aliphatic/aromatic nature. Cys and Pro/Gly have their own classification. It´s important to note that most interactions belong to positively charged aminoacids.

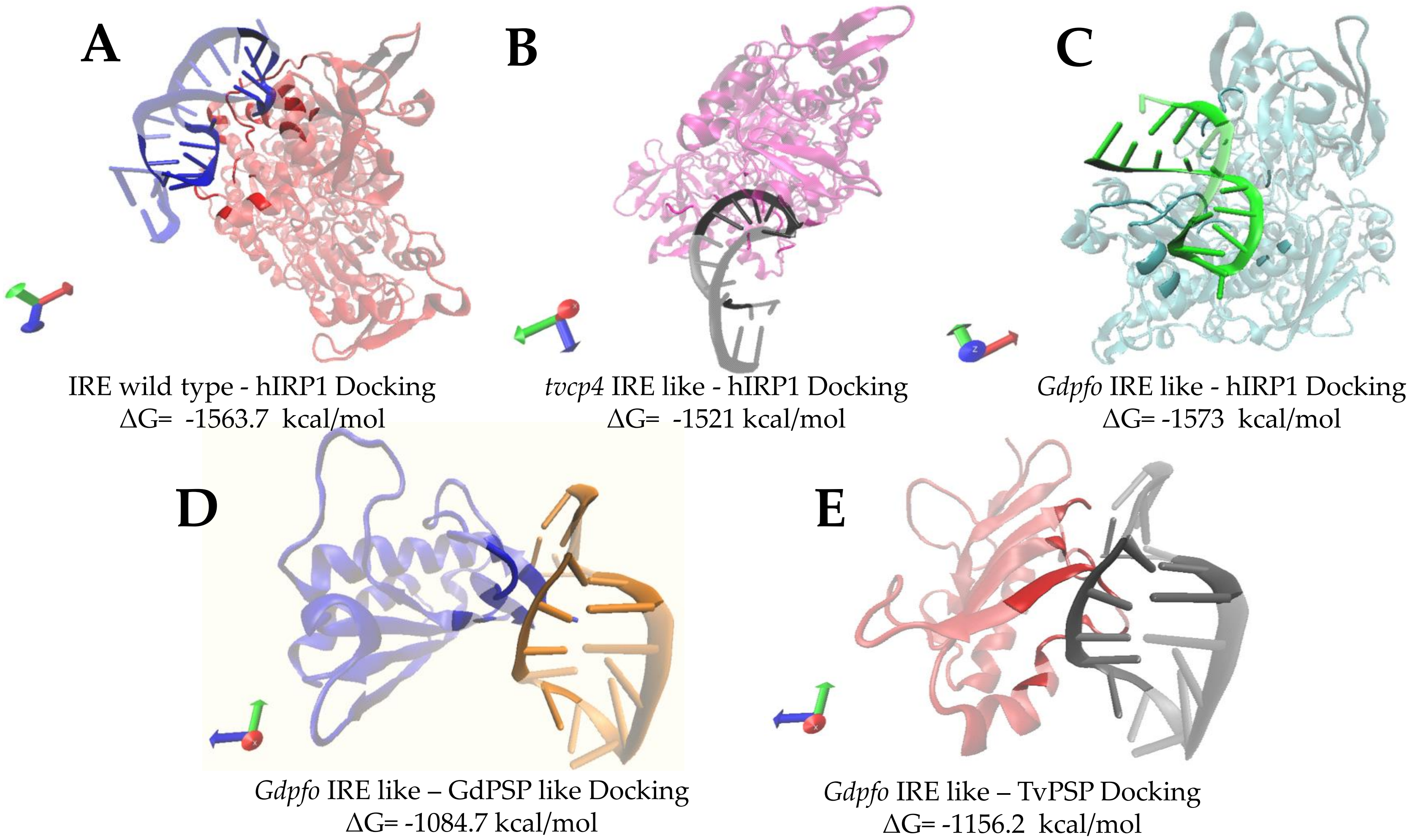

Supplementary Material File S1 (Figure 4). Docking models between IRE and IRE-like structures with hIRP-1 and IRP-like proteins. Highlighted regions in every model represent contact residues. The first ΔG number represents the average of the cluster, and the second number represents the lowest energy. **A.** IRE Wild-type (PDB: 1NBR) is represented by color blue and human hIRP1 (PDB: 2B3X) by red. **B.** *T. vaginalis* cysteine protease 4 (*tvcp4*) IRE-like structure (simulated by simRNA2.0) represented in black and human IRP1 (PDB: 2B3X) by magenta. **C.** *G. duodenalis* pyruvate flavodoxin oxidoreductase (*Gdpfo*) IRE-like structure (simulated by simRNA2.0 (https://genesilico.pl/SimRNAweb)) is represented in green and hIRP1 (PDB: 2B3X) by cyan. **D.** *G. duodenalis pfo* IRE-like structure represented in gray and *G. duodenalis* PSP-like protein (GdPSP-like or Translation Initiation Inhibitor GL50803_00480, TII) in red (modeled with I-TASSER). **E.** *G. duodenalis pfo* IRE-like structure represented in orange and *T. vaginalis* PSP (TvPSP, PDB: 7KGC) in blue. Structures were visualized with VMD (http://www.ks.uiuc.edu/Research/vmd/) and dockings were performed using ClusPro2.0. [118-130].

Supplementary Material File S1, Figure S1 (of Figure 4).

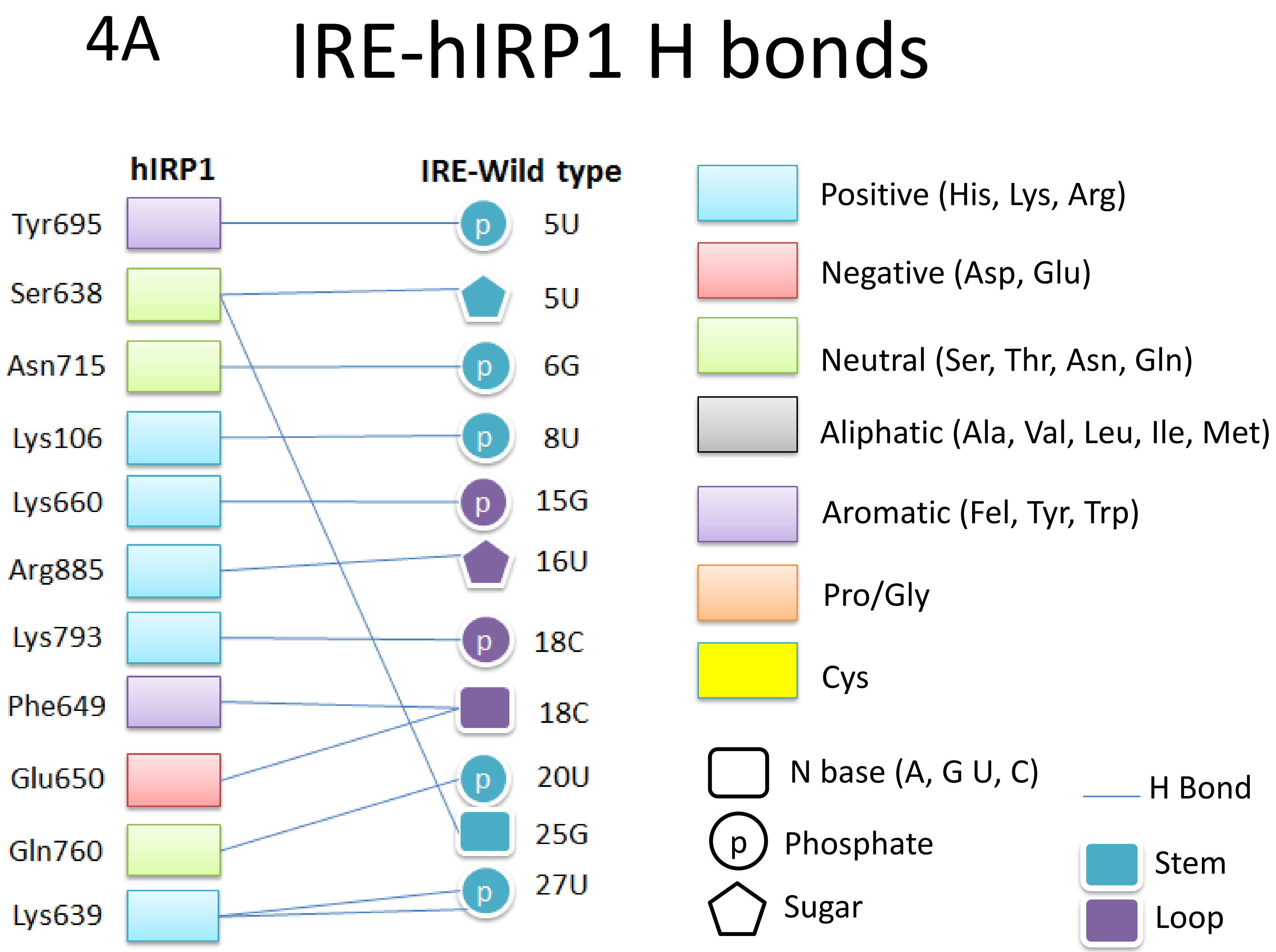

Supplementary Material File S1, Figure S1 (of Figure 4).

4A

hIRP			IRE			Distance
Atom name	Res. name	Res. No.	Atom name	Res. name	Res no.	
OH	TYR	695	OP1	U	5	2.81
O	SER	638	O2	U	5	2.96
ND2	ASN	715	OP1	G	6	2.82
NZ	LYS	106	OP1	U	8	2.80
NZ	LYS	660	OP1	G	15	2.47
NH2	ARG	885	O2	U	16	2.59
NZ	LYS	793	OP1	C	18	2.54
N	PHE	649	O2	C	18	2.84
N	GLU	650	O2	C	18	2.99
NE2	GLN	760	OP1	U	20	2.88
OG	SER	638	H21	G	25	1.93
OG	SER	638	H1	C	26	3.48
NZ	LYS	639	OP1	U	27	2.76
NZ	LYS	639	OP1	U	27	2.76

Supplementary Material File S1, Figure S1 (of Figure 4).

4B *Tvcp4 IRE-like* – hIRP1

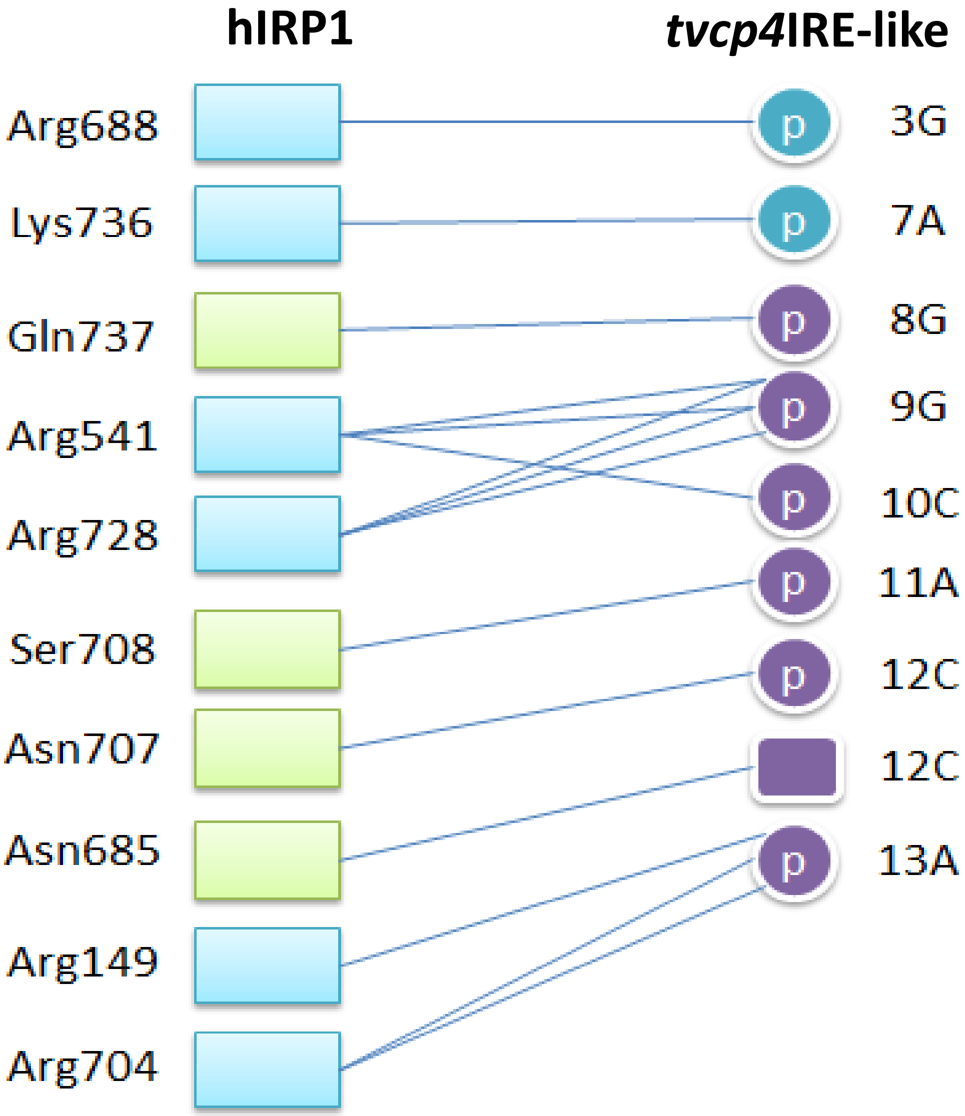

Supplementary Material File S1, Figure S1 (of Figure 4).

4B

hIRP			tvcp4 IRE-like			Distance
Atom name	Res. name	Res. No.	Atom name	Res. name	Res no.	
NH2	ARG	688	OP2	G	3	2.54
NZ	LYS	736	OP2	A	7	2.81
NE2	GLN	737	OP1	G	8	2.89
NE	ARG	541	OP1	G	9	2.96
NH2	ARG	541	OP1	G	9	3.47
N	ARG	728	OP2	G	9	2.96
NH1	ARG	728	OP1	G	9	2.81
NH1	ARG	728	OP1	G	9	2.81
NH2	ARG	541	OP2	C	10	2.73
OG	SER	708	OP2	A	11	2.97
N	ASN	707	OP2	C	12	2.81
ND2	ASN	685	O2	C	12	1.95
NH2	ARG	149	OP1	A	13	2.96
NH2	ARG	704	OP2	A	13	2.74
NE	ARG	704	OP2	A	13	2.88

Supplementary Material File S1, Figure S1 (of Figure 4).

4C *Gdpfo* IRE-like – hIRP1

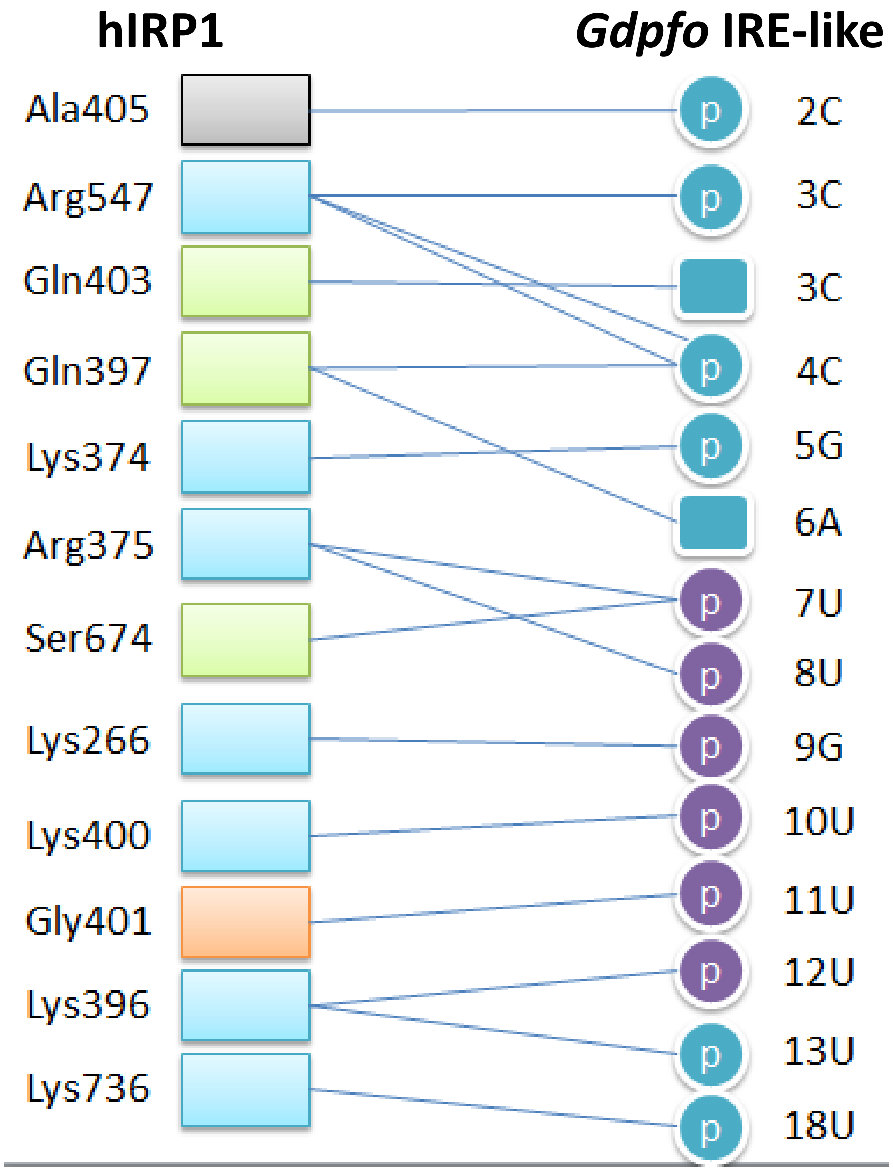

Supplementary Material File S1, Figure S1 (of Figure 4).

4C

hIRP			gdpfo IRE-like			Distance
Atom name	Res. name	Res. No.	Atom name	Res. name	Res no.	
N	ALA	405	OP1	C	2	2.80
NH1	ARG	547	OP1	C	3	2.71
OE1	GLN	397	OP2	C	4	2.70
NH2	ARG	547	OP2	C	4	2.80
NE	ARG	547	OP1	C	4	2.93
NZ	LYS	374	OP2	G	5	2.54
O	GLN	397	N6	A	6	2.67
NH1	ARG	375	OP2	U	7	2.76
OG	SER	674	OP1	U	7	2.94
NH1	ARG	375	OP2	U	8	2.72
NZ	LYS	266	OP1	G	9	2.60
NZ	LYS	400	OP1	U	10	2.68
N	GLY	401	OP1	U	11	2.92
NZ	LYS	396	OP1	U	12	2.81
NZ	LYS	396	OP2	U	13	2.52
NZ	LYS	736	OP1	U	18	2.58

Supplementary Material File S1, Figure S1 (of Figure 4).

4D *Gdpfo* IRE-like- GdPSP-like

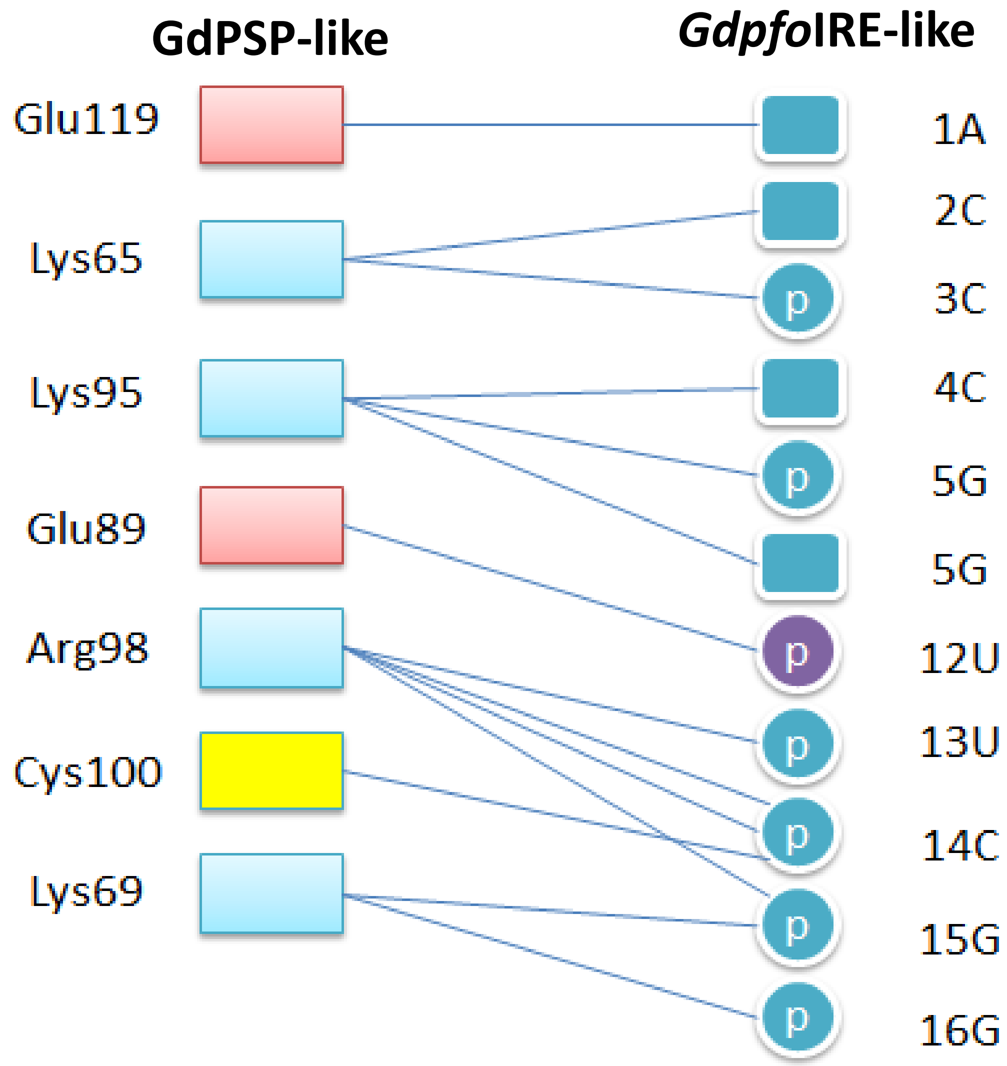

Supplementary Material File S1, Figure S1 (of Figure 4).

4D

GdPSP-like (TII)			gdpfo IRE-like			Distance
Atom name	Res. name	Res. No.	Atom name	Res. name	Res no.	
O	GLU	119	N6	A	1	2.85
O	LYS	65	N4	C	2	2.95
NZ	LYS	65	OP2	C	3	2.80
O	LYS	95	N4	C	4	2.73
NZ	LYS	95	OP2	G	5	2.84
NZ	LYS	95	N7	G	5	2.98
N	GLU	89	OP1	U	12	2.87
NH1	ARG	98	OP1	U	13	2.99
NE	ARG	98	OP2	C	14	2.85
NH2	ARG	98	OP2	C	14	2.87
N	CYS	100	OP1	C	14	2.83
NZ	LYS	69	OP1	G	15	2.62
N	ARG	98	OP2	G	15	2.73
NZ	LYS	69	OP2	G	15	2.62

Supplementary Material File S1, Figure S1 (of Figure 4).

4E *Gdpfo*IRE-like - TvPSP

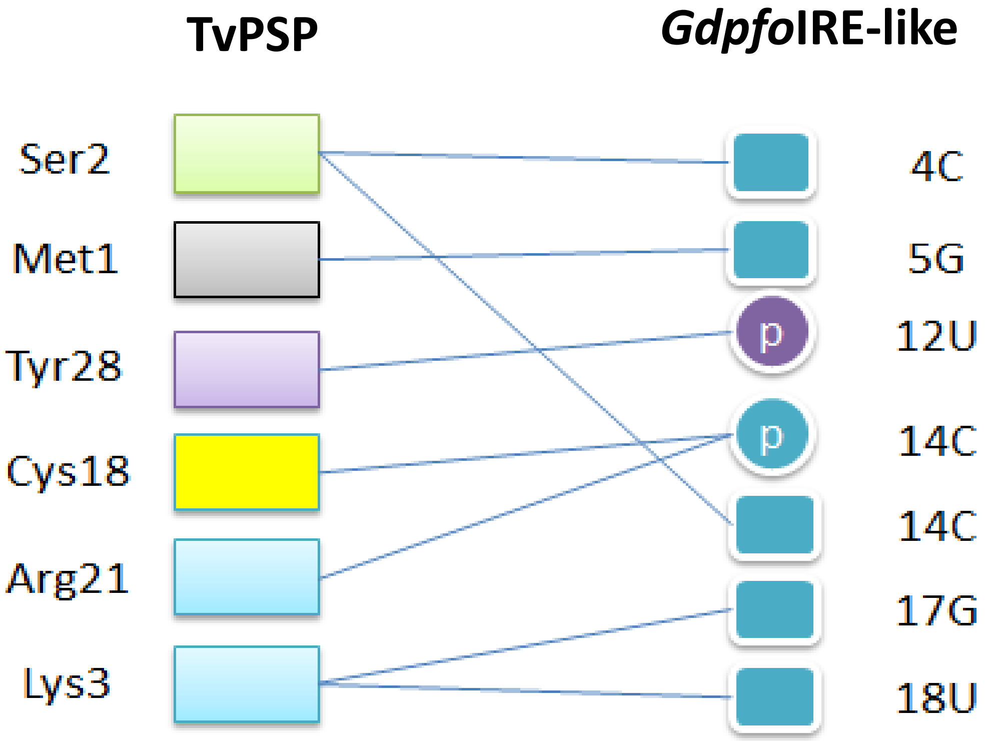

Supplementary Material File S1, Figure S1 (of Figure 4).

4E

TvPSP			gdpfo IRE-like			Distance
Atom name	Res. name	Res. No.	Atom name	Res. name	Res no.	
O	SER	2	N4	C	4	2.61
N	MET	1	O6	G	5	2.93
OH	TYR	28	OP1	U	12	2.81
CB	CYS	18	OP2	C	14	3.94
NE	ARG	21	OP2	C	14	2.54
OG	SER	2	N4	C	14	2.20
NZ	LYS	3	O6	G	17	2.21
NZ	LYS	3	O4	U	18	2.83

Supplementary Material File S1 (Table S1): Possible Iron uptake proteins homologs in *G. duodenalis.*

Supplementary Material File S1. Table S1.

Table S1. Possible Iron uptake proteins homologs in *G. duodenalis*

Used Probe (NCBI)	Homolog Sequence from *Giardia*DB	Access number (*Giardia*DB)	Identity (%)
XP_001317172.1 zinc ion transmembrane transporter protein [*Trichomonas vaginalis* G3]	Giardia Zinc Transporter Protein	GL50803_006664	16.7224
XP_001315331.1 zinc ion transmembrane transporter protein [*Trichomonas vaginalis* G3]	Giardia Zinc Transporter Protein	GL50803_006664	17.4603
XP_649196.1 small GTPase Rab7A [Entamoeba histolytica HM-1:IMSS]	Rab2a	GL50803_0015567	26.2136
XP_649196.1 small GTPase Rab7A [*Entamoeba histolytica* HM-1:IMSS]	Rab2b	GL50803_0016636	27.1845
XP_649196.1 small GTPase Rab7A [*Entamoeba histolytica* HM-1:IMSS]	Rab1a	GL50803_009558	27.6699
